# The Consequences of AMR Education and Awareness Raising: Outputs, Outcomes, and Behavioural Impacts of an Antibiotic-Related Educational Activity in Lao PDR

**DOI:** 10.3390/antibiotics7040095

**Published:** 2018-11-01

**Authors:** Marco J. Haenssgen, Thipphaphone Xayavong, Nutcha Charoenboon, Penporn Warapikuptanun, Yuzana Khine Zaw

**Affiliations:** 1 Centre for Tropical Medicine and Global Health, Nuffield Department of Clinical Medicine, University of Oxford, Old Road Campus, Roosevelt Drive, Oxford OX3 7FZ, UK; 2 CABDyN Complexity Centre, Saïd Business School, University of Oxford, Park End Street, Oxford OX1 1HP, UK; 3 Green Templeton College, 43 Woodstock Road, Oxford OX2 6HG, UK; 4 Mahidol Oxford Tropical Medicine Research Unit, Faculty of Tropical Medicine, Mahidol University, 3/F, 60th Anniversary Chalermprakiat Building, 420/6 Rajvithi Road, Bangkok 10400, Thailand; nutcha7@hotmail.com (N.C.); penporn.wara@gmail.com (P.W.); 5 Department of Peace and Conflict Studies, University for Peace, P.O. Box 138-6100, San José, Costa Rica; thipphaphone37@gmail.com; 6 Department of Political Science, School of Social Science, Ateneo de Manila University, 1108 Metro Manila, Philippines; 7 Lao Oxford Mahosot Wellcome Trust Research Unit (LOMWRU), Mahidol Oxford Tropical Medicine Research Unit (MORU), Faculty of Tropical Medicine, Mahidol University, Bangkok 10400, Thailand; 8 Department of Global Health and Development, London School of Hygiene & Tropical Medicine, London WC1E 7HT, UK; yuzana.khine-zaw@lshtm.ac.uk

**Keywords:** antimicrobial resistance, antibiotics, health behaviour, health education, survey, development studies, rural, LMICs, Lao PDR

## Abstract

Education and awareness raising are the primary tools of global health policy to change public behaviour and tackle antimicrobial resistance. Considering the limitations of an awareness agenda, and the lack of social research to inform alternative approaches, our objective was to generate new empirical evidence on the consequences of antibiotic-related awareness raising in a low-income country context. We implemented an educational activity in two Lao villages to share general antibiotic-related messages and also to learn about people’s conceptions and health behaviours. Two rounds of census survey data enabled us to assess the activity’s outputs, its knowledge outcomes, and its immediate behavioural impacts in a difference-in-difference design. Our panel data covered 1130 adults over two rounds, including 58 activity participants and 208 villagers exposed indirectly via conversations in the village. We found that activity-related communication circulated among more privileged groups, which limited its indirect effects. Among participants, the educational activity influenced the awareness and understanding of “drug resistance”, whereas the effects on attitudes were minor. The evidence on the behavioural impacts was sparse and mixed, but the range of possible consequences included a disproportionate uptake of antibiotics from formal healthcare providers. Our study casts doubt on the continued dominance of awareness raising as a behavioural tool to address antibiotic resistance.

## 1. Introduction

Antibiotic resistance (ABR) as a subset of antimicrobial resistance (AMR) has reached the highest policy levels. National and global policies have been developed to address a problem that is feared to become the leading cause of death globally by 2050, with a disproportionate impact on low- and middle-income countries (LMICs) [1,2]. The leading global policy document to address AMR is the *Global Action Plan on Antimicrobial Resistance* by the FAO/OIE/WHO Tripartite Collaboration on AMR (FAO: Food and Agriculture Organization; OIE: World Organisation for Animal Health; WHO: World Health Organization), the first objective of which is to “improve awareness and understanding of antimicrobial resistance through effective communication, education, and training” [3] (p. 8). This knowledge deficit approach to population health behaviour is problematic for two main reasons [4,5]: First, it disregards other drivers of population health behaviour—for instance health system dysfunctions and inequities, especially in low- and middle-income settings [1,6]—which could render awareness-raising activities ineffective. Second, health communication can have adverse and unforeseen consequences [7,8], and AMR awareness raising is no exception [9]. This can be seen, for example, in the stigmatisation of pig farmers in Denmark, where leaflets had started to advise that, “If you work on a pig farm, you should refrain from having sex with others or seeing anybody […]” [10] (p. 4). These reasons make it questionable as to whether the awareness-raising agenda can live up to its expectations of changing population behaviour in the context of global antibiotic and antimicrobial resistance.

Awareness raising is, of course, not the only global health policy tool used to address ABR and AMR more generally—complementary approaches also involve, for instance, drug research and development, public health interventions, new diagnostic tools, and surveillance activities [2,3,11]. In addition, national-level action to address ABR-related population behaviour has taken more diverse approaches [12], and internal global health policy development processes have started considering interdisciplinary perspectives of human behaviour [13]. However, the awareness agenda remains the central mechanism to address human behaviour on the global policy level, public health research on health communication tends to focus on positive outcomes among intended audiences [8], and the continued underrepresentation of social science research (comprising only 0.6% of all AMR-related publications) impedes a more holistic and nuanced approach to human behaviour in antibiotic and antimicrobial resistance. (There were 345,410 AMR-related publications until 2019. Medicine, biology, immunology, pharmacology, and related disciplines: 83.9%; other disciplines outside the social sciences: 14.7%; social sciences: 0.6%; unspecified “multidisciplinary” publications: 0.9%. Results as of 30 August 2018, based on the Scopus database search query: TITLE-ABS-KEY [“antibiotic resistance” OR “drug resistance” OR “antimicrobial resistance” OR “AMR”] [14]).

This study aimed to inform the awareness agenda from a social sciences perspective by assessing the outputs, outcomes, and behavioural impacts of an ABR-themed educational activity in the low-income setting of Southern Lao PDR. Detailed quantitative health behaviour data, collected in two census survey rounds in two villages, enabled us to document the intended and unintended consequences of awareness raising in a difference-in-difference design.

Although this work did not constitute a formal evaluation, because the research team both developed and assessed the consequences of the educational activity [15], our study design enabled us to detect direct and indirect consequences of the activity—positive as well as negative. Our detailed quantitative health behaviour data, from nearly 2500 survey interviews, permitted us to document the inequitable diffusion of new information, mild changes in villagers’ awareness of drug resistance, weak links to antibiotic-related attitudes, and mixed but potentially detrimental impacts on antibiotic consumption. This study, therefore, contributes to the limited, yet growing, social understanding of the consequences and limitations of ABR interventions in low-income contexts.

## 2. Material and Methods

### 2.1. Study Site

**Figure 1:**
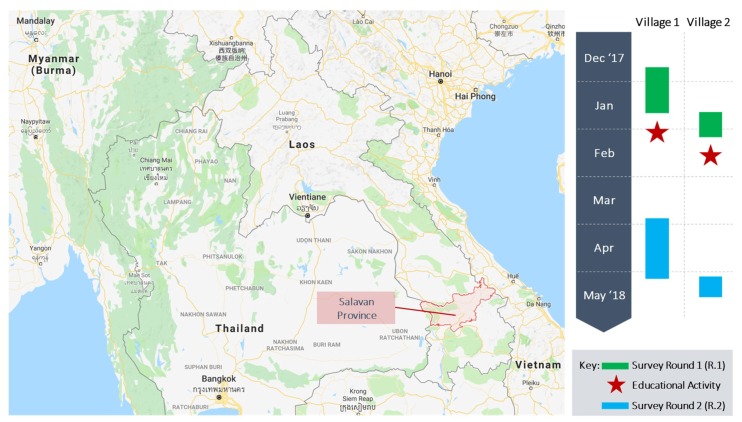
Site map and research timeline in Lao PDR. Source: Authors, adapted from Google Inc. [18].

Our study was part of a research project on rural medicine use in the context of marginalisation in Thailand (Chiang Rai) and Lao PDR (Salavan) [16]. In the present study, we assessed a half-day educational activity that interspersed two rounds of complete census surveys, in two peri-urban villages, near a district capital city in Salavan—Lao PDR’s poorest province (see Figure 1 for a map and timeline of research activities) [17]. The village locations reflected an environment of higher affluence and smaller families compared to the provincial average (Table 1), but we nonetheless observed widespread hardship consistent with the low-income country setting of Lao PDR. The selection of the villages was aided by a local public health official who had indicated interest in these village case studies for future education and communication activities.

### 2.2. Study Population

The study population included Lao villagers aged 18 years old and above. We did not focus specifically on patients but rather on the general population. We excluded respondents who were unable to participate in the study after two attempts to arrange an interview, and we excluded adolescents and children because adults typically acquire and administer antibiotics together with, or on behalf of, children [21].

### 2.3. Intervention (Educational Activity)

We developed the educational activity following more than a year of qualitative research on antibiotic use and treatment-seeking behaviour in Thailand, Myanmar, and Lao PDR. Owing to the public engagement nature of the activity, the development studies background of our research, the caveats in the social sciences literature on health communication, and our own research on antibiotic use and treatment-seeking behaviour in rural Southeast Asia, we decided that the objective of the activity was not to change behaviour, nor to convince villagers that their current behaviour was wrong. The aim of the activity, rather, was to share information and ideas about antibiotics and drug resistance in line with messages from the World Health Organization (e.g., [22]), and also for the team to learn, from the participating villagers, about their antibiotic-related behaviour and conceptions, as well as how they received the messages from the activity. Nevertheless, our two-round data collection enabled a quasi-experimental design in which we could ascertain knowledge, attitude, and behaviour changes among the people who participated in the activity (i.e., direct exposure), who talked about the activity (i.e., indirect exposure), and who were not involved in the activity in any way (i.e., unexposed).

The one-off activity in Salavan was designed to cover half a day of interactive sessions for 25 to 40 people. (We developed the activity simultaneously for rural Thailand and rural Lao PDR as part of the larger project. Because of the cultural context and varying logistical constraints, the specific sessions in the two countries varied slightly [16,23]. For example, the Thai activity included traditional song and a poster-making exercise.) We selected well-known individuals from across each village, expecting that they would inform other villagers about the content of the activity (corresponding to approaches of targeting opinion leaders [24,25]).

The six sessions of the activity (presented in detail in Table 2) covered: A mapping exercise, to understand treatment choices in the village; a medicine matching game, to learn about local conceptions and medicine uses; a resistance game, to introduce the idea of evolving microbes; a role-play activity, to explain drug resistance in a social context; a healthy-wealthy game, to reflect on treatment-seeking choices; and a final feedback and reflection session, to understand how the participants received the messages from the activity. Specifically, the messages embedded in the activity were:

- “Always follow health workers’ advice when using antibiotics”,
- “Never demand antibiotics if health workers say you do not need them”,
- Only use antibiotics when prescribed by a certified health professional”,
- “Germs can become ‘stronger’ if treated inappropriately until the point that there is no medicine to treat them anymore”, and
- “Drug resistance can spread”.

### 2.4. Data Collection

Our survey instrument was a 45-min questionnaire on antibiotic-related attitudes and knowledge, treatment-seeking behaviour, and social networks alongside standard demographic indicators [16]. The questionnaire was administered face-to-face using tablet computers with the SurveyCTO software [26]. Among others, we elicited attitudes and knowledge of antibiotics that corresponded broadly to WHO messages (e.g., [22]), by asking four questions on whether respondents: (a) Would buy antibiotics over the counter, (b) would prefer antibiotics over alternatives, (c) would keep antibiotics for future use, and (d) had considered the fact that antibiotic resistance can spread. Considering the range of possible responses to these questions, we trained our survey teams to field-code the responses as “desirable” or “undesirable” from the point of view of the original WHO messages. (An “undesirable” assessment of behaviour, according to these criteria, did not necessarily mean that people’s behaviours were implausible, irrational, or otherwise inferior; it was merely intended to guide the analysis in light of the objectives of mainstream approaches to awareness raising.) We also collected detailed information on acute illnesses and accidents among the respondents, and/or the children under their supervision, within the two months preceding the survey, and the healthcare choices (healthcare providers, medicine) that the respondents made therein.

### 2.5. Sample Size

We collected two rounds of complete census data from all adult villagers. Satellite images helped to enumerate 578 potentially residential buildings, which comprised 459 households [27]. We defined a household as a group of people sharing a kitchen, and its members as those who had lived in the village for the past six months. The ensuing sample comprised 2480 interviews (1264 in Round I and 1216 in Round II), whereby we interviewed virtually every adult at least once, and 89.4% of the Round I respondents could be re-interviewed (only two households refused participation). Individuals who could only be interviewed once tended to migrate for labour, which is reflected in a larger share of male respondents (54% vs. 46% in the panel) and younger respondents (33.9 years vs. 40.5 years) compared to the panel average. As part of the village population censuses, we recorded 512 completed illness episodes in the first survey round, and 284 in the second. The description of the survey sample is presented in Table 3.

### 2.6. Study Outcomes

The analysis was guided by a simple evaluation framework that considered outputs, outcomes, and impacts of the activity, as shown in Table 4. Note that, because the study team developed and assessed the activity, this did not constitute a formal evaluation. In addition, because we did not specify behaviour change targets, we were more interested in an exploratory analysis of the range of outcomes, be they positive or negative.

### 2.7. Data Analysis

We analysed the quantitative survey data descriptively in a difference-in-difference approach, which enabled us to isolate general trends from changes associated with the educational activity. Because we surveyed all adults in our study villages, and because the educational activity took place between the two survey rounds, we could consider three-month changes of attitudes and behaviours for three groups: People who participated in the educational activity (direct exposure), people who talked about the activity (indirect exposure), and people who neither talked about nor participated in the activity (unexposed).

On the individual level, we used the matched panel data set based on the census data, which allowed like-for-like comparisons of the villages before and after the activity. This enabled us to compare group means before and after the educational activity in each village (first difference), and the difference of these averages between exposed and unexposed groups (difference-in-difference). Because we used complete village census data on the individual level, rather than a sample of the village populations, inferential statistics and confidence intervals were inapplicable and thus were omitted from reporting. In other words, our sample comprised the complete population of the two villages; whereas otherwise the data from two villages would be insufficient to make inferences for the Salavan population. (Another part of the broader research project involved representative survey data collection to enable inferences for the provincial population.)

Data on the illness level could not be matched accordingly, considering that only a subset of respondents would report an acute illness or accident, which were also not immediately comparable for the same individual across the two survey rounds. As a result, we treated the data on the illness level as repeated cross-sectional data rather than panel data. The practical implication was that the interpretation of our descriptive analysis of immediate behavioural impacts had to be more cautious. (Multilevel models in future research will account for individual-level clustering of behaviour.) Owing to the smaller size of the illness samples across the three study groups, we also carried out sensitivity analyses of antibiotic use, from formal and informal sources, using respondents’ attitudes towards buying antibiotics over the counter (see Supplementary Materials, Table S2). For illustrative purposes, we used Wilcoxon rank-sum hypothesis tests for non-normally distributed variables [28,29]. The analysis was carried out using Stata 15 [30].

### 2.8. Ethical Considerations

The research was reviewed and approved by the University of Oxford Tropical Research Ethics Committee (Ref. OxTREC 528-17), and it received local ethical approval in Thailand from the Mae Fah Luang University Research Ethics Committee on Human Research (Ref. REH 60099), and in Lao PDR from the National Ethics Committee for Health Research (Ref. NEHCR 074). Permission to access the study villages was obtained from local security authorities and villages leaders. Participation in the interviews and educational activity was voluntary and informed verbal consent was obtained from all study participants, which was audio recorded and documented in a written record of oral consent by the survey field investigators for each participant. The participants received a small financial token of appreciation of the equivalent of GBP 1.00 for the survey, and of the equivalent of GBP 3.00 for the educational activity.

## 3. Results

### 3.1. Outputs

The educational activity took place in two comparatively large peri-urban villages, located on main roads with easy access to urban and formal health facilities. Within the villages, a range of informal healthcare providers, including traditional healers and grocery shops, provided treatment and medicine alongside public and private primary healthcare providers.

The activity was attended by approximately 30 people per village and implemented in the presence of local officials (i.e., the village head and the public health officer). Most participants in both villages were highly engaged throughout the day, and the presence of village administration and medical officers throughout the entire activity did not appear to inhibit the participation of the villagers. They eased into discussing and exchanging ideas with their team mates particularly during small group sessions, but they were more hesitant to share their discussions in the larger group. Nevertheless, through the sharing of their ideas, the study team learned that the participants were familiar with common medicines. In the “medicine matching” session, the villagers categorised antibiotics such as capsules of penicillin or ampicillin commonly in a single group, whereas other medicines were grouped by their mode of administration (e.g., eating, diluting in water, injection, etc.), their shape (e.g., tablets, liquids, etc.), and their function (e.g., for coughing, headaches, etc.). Our final feedback and evaluation session revealed, further, that the respondents were able to identify the key messages that we intended to share. For example, one participant responded that she would tell her husband to stop buying and keeping antibiotics at home. However, the feedback session involved only a small sample of three to four participants per village. Therefore, we based the remaining analysis of the activity outcomes on our quantitative data.

**Figure 2:**
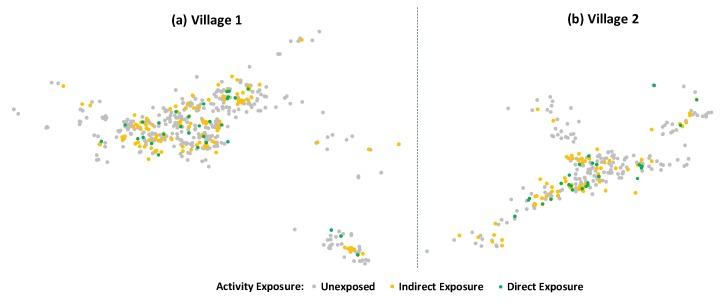
Map and overview of educational activity exposure in case study villages. Notes: “Endline” (R.2) data, using matched panel data. *n* = 723 (Panel (**a**)), *n* = 407 (Panel (**b**)). Marker size adjusted to distinguish overlapping responses.

The characteristics of the 58 participants are presented in Table 5, together with 208 respondents who talked about the activity in the village, and 864 villagers who had not been exposed directly or indirectly (also mapped in Figure 2). The table indicates that the activity participants included a higher share of women and higher levels of education than the village average. People who talked about the activity also tended to be female and better educated, whereas the small fraction of non-Lao ethnic groups remained largely unexposed.

**Figure 3:**
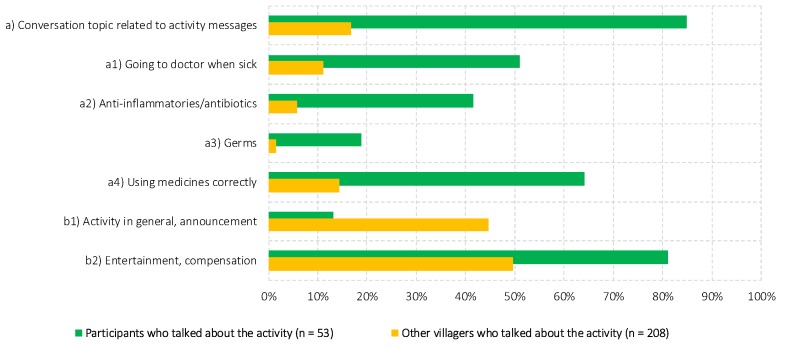
Differences in communicated activity themes across participants and non-participants. Notes: “Endline” (R.2) data, using matched panel data.

As the maps in Figure 2 above indicated, indirect exposure was widespread but less likely to permeate peripheral areas of the villages. In addition, indirect exposure encapsulated only a fraction of the themes of the educational activity, as Figure 3 demonstrates. More than 90% of the participants reported conversations with other villagers about the activity, 85% of whom recalled conversation themes that related directly to the activity content (e.g., going to the doctor when sick). Another common theme, among 81% of the participants, was the entertainment component of the activity. In contrast, among villagers who did not participate in the activity, conversation themes were almost exclusively limited to the activity in general (e.g., the activity being announced by the village head) and to its entertainment component. Only 17% of the 208 indirectly exposed villagers recalled a theme that related to the antibiotic content of the activity. The striking mismatch between sent and circulated themes suggested that indirect exposure to the activity content was limited, despite the extensive conversations across the village. Because the villagers who recalled activity-related themes were on average slightly wealthier, by 0.055 wealth index points (0.581 vs. 0.526), and had two more years of formal education (9.9 vs. 7.8 years), content-related indirect exposure also appeared to have been confined to socio-economically more privileged strata. In the next section, we examine the outcomes of the direct and indirect exposure on awareness and interpretations of “drug resistance”, and on villagers’ antibiotic-related attitudes and knowledge.

### 3.2. Outcomes

Table 6 summarises the outcomes of the activity, reporting knowledge and attitudes, and is grouped by directly, indirectly, and unexposed groups. For each group, we report average knowledge and attitudes before and after the educational activity, together with the before–after difference, and difference-in-difference statistics comparing exposed to unexposed group differences. Graphs of the outcomes reported in this section are presented in Supplementary Materials, Figures S1–S3.

The outcomes of the activity were concentrated on the knowledge and attitudes among people who were exposed directly. The recognition of the various terms for “drug resistance” is documented in Table 6, Section a1, for the technical but lesser-known term of “due yah” (literally translated as “stubborn [to the effects of] medicine”) and Section a2, for the colloquial but broader translation “lueng yah” as meaning “[e.g., the body getting] used to medicine”. Against the generally increasing trend in the recognition of “due yah”, the recognition of the term increased disproportionately among the participants of the activity—at least by 22.6 percentage points across all groups, which is potentially an artefact of the survey asking the same question twice in three months. The average recognition of the term rose from 27.6% to 91.4% among the participants, compared to 36.2% to 58.8% among the unexposed group. In contrast, the colloquial expression of “lueng yah” was already widely recognised prior to the educational activity (at least 80.2%), and direct and indirect exposure exhibited only a marginally higher rate of change in the first village compared to the unexposed group.

Even in the absence of changing rates of recognition, the educational activity might have influenced people’s interpretation of the term “drug resistance”. For both notions (“due yah” and “lueng yah”), we present the interpretations in Table 6, Sections a1 and a2 (Responses to the question, “What do you think is ‘drug resistance?’”). Section a1 shows that the interpretation of “due yah” changed the most with the participants of the activity, where a decreasing share of “don’t know” responses (−31.0%) was replaced with broad and antibiotic-independent interpretations of “due yah means lueng yah” (+29.3%), “medicines, in general, do not work anymore” (+10.3%), and “taking medicines in the wrong way” (+5.2%). The activity participants also exhibited fewer interpretations that conflict with the biomedical concept of drug resistance, like “patients being stubborn” (−3.5%), “being addicted to medicine” (−3.5%), or “medicines having side-effects” (−6.9%). (Side-effects here refer to medicine-related complications such as dizziness, nausea, rashes, or allergic reactions, not to drug resistance.) However, the fraction of interpretations relating directly to antibiotics and germs only increased marginally, from 1.7% to 3.5%, among the participants, and it decreased more strongly among indirectly exposed villagers than among the unexposed group (from 5.3% to 1.0% vs. from 3.7% to 2.4%, respectively). Aside from an increase in the interpretation “due yah means lueng yah” (+24.0%), the indirectly exposed group also exhibited small changes in the themes “stubborn patients” (−6.3%) and “medicine side-effects” (+2.9%). Section a2 in Table 6 indicates that changes in the interpretation of “lueng yah” were less pronounced among all groups. Compared to the trend among unexposed villagers, activity participants, nevertheless, declared more interpretations relating to antibiotics and drug-resistant germs (+3.4%), and fewer interpretations relating to “drug resistance” as medicine addiction (−10.4%). Among the indirectly exposed group, we also observed a shift away from antibiotic-specific (−11.1%) towards more general interpretations of medicine becoming less effectual (+8.7%; typically involving notions that the body develops a “tolerance” against medicine in line with the broader meaning of “lueng yah”).

Despite the association between the activity and villagers’ recognition and interpretation of “drug resistance”, the changes in the biomedical “desirability” of people’s antibiotic-related knowledge and attitudes varied to a small extent only across the study groups. As reported in Section b of Table 6, direct exposure yielded a slightly stronger increase in our assessment of “desirability” (+0.10 index points on a scale from 0 to 4), whereas indirect exposure was not associated with an increase beyond the general trend (−0.14 vs. unexposed trend of −0.11). Broken down by its four component questions, the sources of the slightly stronger increase among the directly exposed group were the attitude to not buy antibiotics over the counter (from 43.8% to 55.2%; disproportionately large increase compared to other groups), and the knowledge to not keep antibiotics at home for future use (from 27.6% to 32.8%; an increase higher than the unexposed group but short of the larger increase in the indirectly exposed group). Curiously, responses to the question “Can your ‘due yah’ spread to other people?” became noticeably less “desirable” over time among the groups that either had indirect exposure or no exposure to the activity (−13.9% and −9.5%, respectively). Overall, the outcomes on attitudes are inconclusive despite the mild relative increase in our measure of “desirability” among the activity participants.

### 3.3. Impact

**Figure 4:**
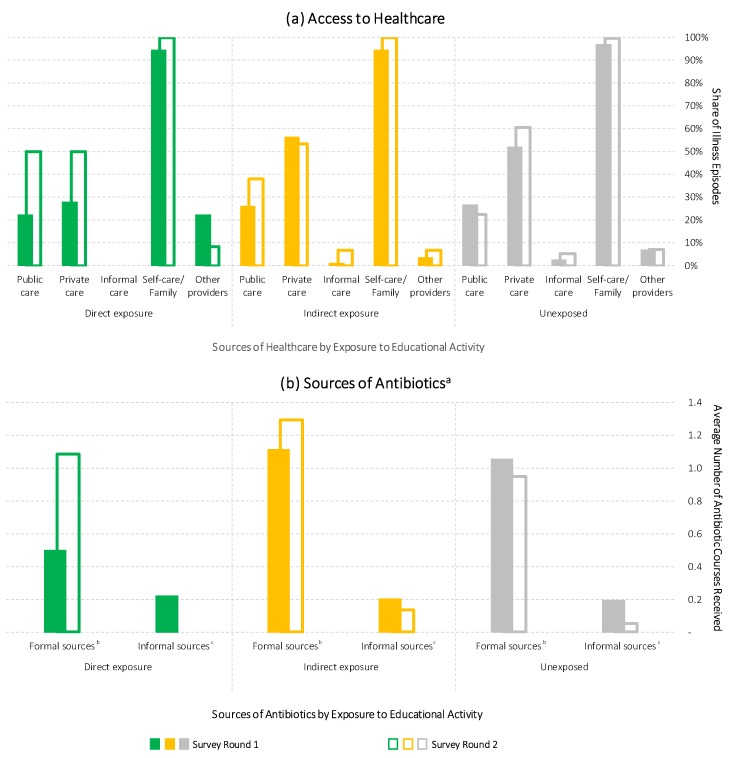
Healthcare access and sources of antibiotics across survey rounds. Panel (**a**) Patterns of healthcare access by activity participation. Panel (**b**) Sources of antibiotics across survey rounds. Notes: Pooled data set, using complete survey data; repeated cross-sections of illness episodes (*n* = 727). Sub-sample sizes by survey round are: Round 1/direct exposure (*n* = 18)/indirect exposure (*n* = 89)/unexposed (*n* = 337); Round 2/direct exposure (*n* = 12)/indirect exposure (*n* = 59)/unexposed (*n* = 213). Multiple sources of antibiotic access per illness episode were possible. ^a^ “Antibiotics”, including confirmed and possible antibiotics, were based on reported medicines received during the illness, and respondent’s reported names and uses of antibiotics shown during the interview. ^b^ Includes public and private healthcare providers. ^c^ Includes traditional healers, grocery stores, retired doctors, itinerant medicine traders, and medicine stored at home and provided by family and friends.

As the final step in our analysis, we examined the impact of the activity on villagers’ behaviour, focusing specifically on healthcare choices and antibiotic use during recent acute illnesses and accidents. The main results are presented in Figure 4 and detailed results are in Supplementary Materials, Table S1.

Panel a in Figure 4 presents the patterns of healthcare access during people’s recent illness episodes with the caveats that (a) people could report multiple healthcare choices during an illness episode, (b) illnesses in Survey Round 2 were reported less frequently, (c) the self-reported severity of illnesses was marginally higher in Survey Round 2 (see Supplementary Materials, Table S1 for details), and (d) the illness episode samples among the directly exposed group were small, with 18 and 12 observations in Survey Rounds 1 and 2, respectively, and should therefore be considered cautiously. Pending further discussion in the next section, the main observations from this panel were:

- The directly and indirectly exposed groups reported higher public healthcare access in the second survey round.
- Private healthcare access followed a slightly increasing trend that was absent from the indirectly exposed group.
- Informal healthcare access was generally low but followed a slightly increasing trend that was absent from the directly exposed group.
- The involvement of “other” healthcare providers (especially retired doctors running informal practices in the villages) decreased only in the directly exposed group—to a level similar to the other groups.

While these patterns suggest that there may be an association between the educational activity and the seeking of public healthcare, Panel b explores in greater depth the use of antibiotics from formal and informal healthcare sources (including both confirmed antibiotics and unclassified medicines that might be antibiotics). Contrary to the patterns of informal healthcare access, reported antibiotic use from informal sources was consistently lower across all three groups in Survey Round 2. In addition, whereas the unexposed group indicated a slight general decline in antibiotic use from formal sources, the directly and indirectly exposed groups exhibited higher rates in Survey Round 2 (which exceeded the lower informal antibiotic use). At the same time, formal antibiotic access among the activity participants rose to a level similar to the first-round levels in the other two groups, which suggests regression to, and random variation around, the mean, rather than an actual effect of the educational activity. Therefore, despite the possible trend, the impact of the activity on antibiotic use remained inconclusive.

## 4. Discussion

Our educational activity had geographically widespread direct and indirect exposure, but closer examination of communication patterns revealed that activity-related messages did not circulate within the villages as a whole but rather circulated within socio-economically more privileged strata. The educational activity entailed changes in antibiotic-related attitudes and in awareness and interpretation of the term “drug resistance”, which were broadly in line with the content of the activity but limited to the directly exposed participants. Weak outcomes among indirectly exposed groups could be explained with the aforementioned communication frictions. Furthermore, the association between the activity and health behaviour was weak at best, and at worst it could suggest that activity participants overcompensated antibiotics from informal sources with antibiotics from formal healthcare providers.

Three study limitations deserve particular discussion. Firstly, the extensive presence of the survey teams and participation in the activity could have contributed to priming and social desirability biases. However, our difference-in-difference design mitigated the influence of social desirability, and the feedback from the villagers, and from regular survey team meetings, suggested that social desirability played a small but not overwhelming role in explaining people’s responses.

Secondly, the small sample of illness episodes among the activity participants weakened the analysis, and rendered the impact assessment indicative only. Our sensitivity tests (Supplementary Materials, Table S2) suggested that informal antibiotic use was consistently lower among people with “desirable” attitudes, but this reduction could be outweighed by formal antibiotic use depending on the definition of antibiotics. More specifically, we used Wilcoxon rank-sum hypothesis tests [28,29] to compare antibiotic use among villagers with “desirable” and “undesirable” attitudes towards buying antibiotics over the counter. The comparison suggested that people with more “desirable” attitudes had significantly higher use of confirmed antibiotics from formal sources (*p* < 0.10), which outweighed the lower use of antibiotics from informal sources (*p* < 0.05). If unclassified medicines were also included in the definition of medicines that could possibly be antibiotics, then the informal antibiotic use among people with more “desirable” attitudes was still significantly lower (*p* < 0.01), but outweighed the small and statistically insignificant difference between antibiotics from formal sources. The results therefore remained inconclusive but reinforced the possibility that more “desirable” attitudes could needlessly increase antibiotic use.

Thirdly, our design choices (peri-urban setting, purposive participant selection) limit the external validity of our findings. Our larger research project on rural health behaviour in Salavan and Chiang Rai is nevertheless able to put our findings into perspective. For example, our representative survey in rural Salavan documented a high formal antibiotic use and a similarly weak link to antibiotic-related attitudes [31], and a similar educational activity in Chiang Rai included side-effects such as rumours and increasing informal healthcare access [23]. The comparatively small side-effects in Salavan may have resulted from programmatic choices and contextual variation, like the presence of health officials and the peri-urban environment.

In summation, this study suggests that the outputs of our educational activity diffused inequitably, its outcomes on awareness were discernible whereas the effects on attitudes were weak, and its immediate behavioural impact was inconclusive with potentially detrimental facets. Together with the residual influence of social desirability biases, this case study adds to the social understanding of awareness-raising health interventions, and provides support for the caution with which the social sciences approach health communication, in general, and AMR-related awareness raising, in particular.

It appears that one obstacle—perhaps obvious for medical sociologists—to changing behaviours was that people had existing notions around drug resistance as a vernacular concept. Rather than being “empty vessels” [32], new information competed with people’s existing knowledge, and other sources of information [33]. Beyond the activity participants, the limited circulation of content-related themes suggested that the main messages resonated with more privileged lifestyles irrespective of our efforts to translate and convey them in a way that is meaningful for the broader rural population (similar to core-periphery tensions raised by Broom, et al. [34]). A further complication for the diffusion of the messages was that people may not deem information about drug resistance a personal priority [4,35] (we do not take a stance here as to whether people “should” see AMR as a personal priority), and the benefit of slowing the development of drug resistant bacteria lacks a clear demonstration effect [24]. If these arguments hold, then new ideas about antibiotic use would remain passive unless they threaten people personally (or we would need to combine the content with “a spark”). However, other authors have cautioned against backlashes from fear-based AMR narratives [10,36]. Problems like adverse behavioural reactions, stigma, or public resentment may be accentuated even further in situations like ours, where antibiotics cannot be easily identified and people might not actively distinguish between antibiotics and other types of pharmaceuticals [37].

## 5. Conclusions

We developed an educational activity and deployed it, between two rounds of complete adult population censuses, in two peri-urban villages in Salavan. Difference-in-difference analysis of the survey data provided a detailed picture of the activity’s outputs, outcomes, and impacts to inform awareness-centric global AMR agendas.

The two-directional educational activity enabled us to learn about the medicine use of the participating villagers in peri-urban Salavan, and it permitted us to share antibiotic-related ideas and messages, albeit their outcomes on attitudes and their immediate behavioural impacts were limited. As an antibiotic-related awareness-raising intervention at scale, our approach would face obstacles. On the one hand, the small-group format of our educational activity does not lend itself to deployment among an entire village population. On the other hand, the incomplete diffusion of the messages beyond the participants suggested that the beneficiaries of the activity would be more privileged groups. Other forms of awareness raising, such as hospital- or mass-media-led information campaigns, may be able to reach out further, but they, too, may suffer from inequitable uptake and unforeseen interpretations of messages across socio-economic strata.

Still, no matter how encouraging the awareness-raising outcomes were, the weak and/or ambiguous link between awareness, attitudes, and behaviour should lower our expectations about antibiotic-related awareness raising to change treatment-seeking behaviour. Existing behaviour may rather be driven by such factors as personal experience, advice, help from family members and friends, despair, and/or uncertainty in an obscure and fragmented health system [38,39,40,41,42]. The continued high level of antibiotic use among participants and villagers with already “desirable” attitudes, together with widespread poverty and the generally low access to public healthcare, even in our peri-urban setting, suggest that solutions to problematic forms of antibiotic use do not necessarily reside in the domain of awareness raising, but rather in more fundamental areas like access to healthcare and medicine. Our case does not render awareness-raising activities obsolete, but it does suggest that they can, at best, be only a small facet of AMR-related behavioural policies.

## Figures and Tables

**Table 1:** Characteristics of survey villages compared to the provincial average.

Village Attributes	Village 1	Village 2	Salavan Average
Village Size	1462	744	369 ^a^
Household Size	5.0	4.5	5.9
Female Population Share	48.8%	53.1%	50.1%
Dependency Ratio ^c^	0.68	0.63	0.64 ^b^
Households Owning Mobile Phones	96.0%	89.9%	81.6%

Source: Primary survey data and Lao Statistics Bureau [19]. ^a^ Village numbers based on data from National Geospatial-Intelligence Agency [20]. ^b^ National average for rural areas. ^c^ Non-working-age population divided by working-age population (15–64 years).

**Table 2:** Elements of the educational activity.

Session & Duration	Description	Expected Outcomes	Main Message ^a^	Direction of Communication
**Ice Breaking (15 min)**	Ice breaking.			
**(1) Community Mapping (30 min)**	In groups, participants sketch a village map and mark down their own houses, important locations in the village, and draw lines to connect themselves with places as well as people they go to when sick.	Team learns about places, existing health networks, and health facilities within and nearby the village.		Participants ↓ Team
**(2) Medicine Matching (30 min)**	Part (I): Participants sort medicines into 2 groups—those that they know and do not know.	Team gains overview of medicines and their purposes from participants’ perspective.		Participants ↓ Team
	Part (II): Participants free-sort pictures of common medicines into their own categories.	Team understands participants’ general conceptions around medicines and treatments.		Participants ↓ Team
	Part (III): Participants sort medicines into two groups—over-the-counter medicines and prescription medicines.	Participants reflect on the ways to access medicines.	Only use antibiotics when prescribed by a certified health professional.	Team ↓ Participants
**(3) Resistance (30 min)**	Participants pass a germ around in a circle. When the music stops, the person with the germ answers a right-or-wrong question about taking medicines. If incorrect, she/he are out of the game, the germ evolves, and the game continues. The last remaining person wins a prize.	Participants become familiar with the idea of bacteria evolving and resisting medicines.	(1) Germs can become “stronger” if treated inappropriately until the point that there is no medicine to treat them anymore. (2) Drug resistance can spread.	Team ↓ Participants
**Break (15 min)**	Break.			
**(4) Role-Play (20 min)**	A short skit performed by the team with a simple storyline around antibiotics and antibiotic resistance.	Participants gain a deeper understanding about drug resistance and their own part in the issue.	(1) Always follow health workers’ advice when using antibiotics. (2) Never demand antibiotics if health workers say you don’t need them. (3) Drug resistance can spread.	Team ↓ Participants
**(5) Healthy-Wealthy Game (30 min)**	Participants simulate running a family business that produces goods and sells to the market. Each group (family) has different tools to make as much money as they can. Throughout the activity, family members are diagnosed randomly with a disease, provided with different treatment scenarios, and the rest of the family have to pay hospital fees to bring that sick member back.	Participants reflect on common illnesses and the various ways of treating them; the team gains an understanding of health decisions in the local context.	Only use antibiotics when prescribed by a certified health professional.	Team ↓ ↑ Participants
**(6) Feedback and Reflection (15 min)**	Participants provide their reflections on the activities and lessons learned.	Participants understand the key messages from the activities, and express these to the group.		Participants ↓ Participants/Team

^a^ Messages based on World Health Organization recommendations (WHO) (e.g., [22]).

**Table 3:** Sample characteristics of two rounds of census surveys in two Lao villages.

Variable		Survey Round I					Survey Round II				
		Mean	Std. Dev.	Min	Max	*n*	Mean	Std. Dev.	Min	Max	*n*
**Activity**	% participated in educational activity: Throughout	n/a ^g^					0.05	0.21	0	1	1216
	% participated in educational activity: Partly	n/a ^g^					0.00	0.06	0	1	1216
	% heard about educational activity	n/a ^g^					0.22	0.41	0	1	1216
**Demographic Attributes**	Sex (% female)	0.55	0.50	0	1	1264	0.56	0.50	0	1	1216
	Age	39.91	17.14	18	100	1264	40.04	17.02	18	100	1216
	Completed years of formal education	6.28	4.59	0	21	1264	6.22	4.56	0	21	1216
	Wealth index (range: 0 to 1) ^a,b^	0.49	0.13	0.11	0.78	454	0.50	0.13	0.11	0.78	446
	% speaking Lao	1.00	0.00	1	1	1264	1.00	0.00	1	1	1216
	Ethnic group: Lao Loum	0.97	0.18	0	1	1264	0.96	0.19	0	1	1216
	Ethnic group: Other	0.01	0.10	0	1	1264	0.01	0.10	0	1	1216
	Ethnic group: Don’t know/prefer not to say	0.02	0.15	0	1	1264	0.03	0.16	0	1	1216
**Antibiotic Knowledge/Attitudes**	% have seen antibiotic capsules	0.97	0.18	0	1	1264	0.96	0.19	0	1	1216
	% have heard of drug resistance (“due yah”) ^c^	0.39	0.49	0	1	1264	0.63	0.48	0	1	1216
	% have heard of drug resistance (“lueng yah”) ^c^	0.79	0.40	0	1	1264	0.82	0.39	0	1	1216
	% buy antibiotics over the counter (attitude)	0.30	0.46	0	1	1264	0.27	0.44	0	1	1216
	% prefer antibiotics over alternatives (attitude)	0.29	0.45	0	1	1264	0.24	0.43	0	1	1216
	% do not keep antibiotics for future use (knowledge)	0.22	0.41	0	1	1264	0.27	0.44	0	1	1216
	% antibiotic resistance can spread (knowledge)	0.12	0.32	0	1	1264	0.02	0.15	0	1	1216
	No. of desirable knowledge/attitude answers (0–4)	0.93	0.93	0	4	1264	0.81	0.87	0	4	1216
**Treatment-Seeking Behaviour ^d,e^**	% of illness episodes involving children	0.39	0.49	0	1	512	0.35	0.48	0	1	284
	Self-rated severity (1 = mild, 2 = medium, 3 = severe)	1.79	0.70	1	3	512	1.88	0.67	1	3	284
	Average duration of illness episode (days)	7.29	9.25	1	130	512	7.36	14.42	1	219	284
	Average no. of medicines and treatments received ^f^	2.74	1.71	0	13	512	2.46	1.39	0	8	284
	Average no. of antibiotics	0.50	0.70	0	4	512	0.42	0.59	0	3	284
	Average no. of antibiotics (incl. “uncertain” medicine)	1.31	1.45	0	10	512	1.15	1.25	0	6	284
	% public providers (health centres, hospitals)	0.27	0.44	0	1	512	0.27	0.44	0	1	284
	% private providers (clinics, hospitals, pharmacies)	0.53	0.50	0	1	512	0.59	0.49	0	1	284
	% informal providers (grocery stores, healers)	0.02	0.14	0	1	512	0.05	0.22	0	1	284
	% family and self-care	0.97	0.18	0	1	512	1.00	0.06	0	1	284
	% others	0.06	0.25	0	1	512	0.07	0.26	0	1	284

^a^ Average of 17 household assets and amenities on scale from 0 to 1. ^b^ Household-level data. ^c^ The term “drug resistance” has two local expressions: “ດື້ຍາ” (“due yah”) is the formal translation of “drug resistance” and literally translates into “stubborn [to the effects of] medicine”. “ລຶ້ງຍາ” (“lueng yah”) is a colloquial but also broader expression that does not exclusively refer to drug resistance, meaning “[e.g., the body getting] used to medicine”. ^d^ Illness-level data. ^e^ Completed illnesses experienced by a respondent or a child under their supervision. ^f^ “Number of courses” as in, “how many types of medicine did you receive during step x of your illness?” ^g^ Educational activity took place after Survey Round I, therefore no exposure reported.

**Table 4:** Evaluation framework for analysis of study outcomes.

Framework Element	Outputs	Outcomes	Impacts
Level of Analysis	Individual	Individual	Illness
Indicators	Direct and indirect exposure to educational activity	Awareness and understanding of drug resistance ^a^	Patterns of healthcare utilisation during acute illnesses ^c^
	Lessons and feedback from educational activity	“Desirability” of antibiotic-related attitudes and knowledge ^b^	Sources of antibiotics during acute illnesses ^d^

^a^ The term “drug resistance” has two local expressions: “ດື້ຍາ” (“due yah”) is the formal translation of “drug resistance” and literally translates into “stubborn [to the effects of] medicine”. “ລຶ້ງຍາ” (“lueng yah”) is a colloquial but also broader expression, meaning “[e.g., the body getting] used to medicine”. While “lueng yah” did not exclusively refer to drug resistance, it had arisen consistently as a theme during the questionnaire testing phase and, as can be seen in Results Section 3.2, it was commonly mentioned as an explanation for the formal term “due yah”. ^b^ See Table 3 for associated indicators. ^c^ Focusing especially on formal and informal healthcare providers. Formal providers included public hospitals and primary care units (public), and private clinics, private hospitals and pharmacies (private). Informal providers included traditional healers, grocery stores, retired doctors, and itinerant medicine traders. ^d^ Same as above, plus antibiotics stored at home and provided by family and friends as “informal” sources.

**Table 5:** Characteristics of individuals by activity exposure.

Variable	Direct Exposure (i.e., Participated in Activity) (*n* = 58)	Indirect Exposure (i.e., Talked About Activity) (*n* = 208)	Unexposed (*n* = 864)
	Mean (Std. Dev)	Mean (Std. Dev)	Mean (Std. Dev)
Sex (% female)	0.71 (0.46)	0.63 (0.49)	0.54 (0.50)
Age	44.76 (11.36)	38.18 (14.91)	40.85 (17.75)
Education	7.10 (4.06)	8.18 (4.99)	5.67 (4.36)
Wealth index (range: 0 to 1) ^a^	0.51 (0.12)	0.54 (0.11)	0.50 (0.12)
Ethnic group: Lao Loum	1.00 (0.00)	0.99 (0.12)	0.96 (0.20)
Ethnic group: Other	0.00 (0.00)	0.01 (0.10)	0.01 (0.11)
Ethnic group: Don’t know/prefer not to say	0.00 (0.00)	0.00 (0.07)	0.03 (0.16)

Notes: “Endline” (R.2) data, using matched panel data. Groups are mutually exclusive, for example, the group “talked about activity” does not include participants of the activity (among whom 53 talked about the activity with other villagers). ^a^ Average of 17 household assets and amenities on scale from 0 to 1.

**Table 6:** Antibiotic-related attitudes and knowledge across survey rounds.

Variable		Direct Exposure (*n* = 58)			Indirect Exposure (*n* = 208)			Unexposed (*n* = 864)			Difference-in-Difference	
		Survey Round 1	Survey Round 2	Diffe-rence	Survey Round 1	Survey Round 2	Diffe-rence	Survey Round 1	Survey Round 2	Diffe-rence	Direct vs. Unexposed	Indirect vs. Unexposed
**(a1) Awareness of Drug Resistance (Due Yah)**	**Heard of drug resistance (“due yah”)**	27.6%	91.4%	+63.8%	49.0%	75.0%	+26.0%	36.2%	58.8%	+22.6%	+41.2%	+3.4%
	**Interpretations:** Reference to antibiotics/drug-resistant germs	1.7%	3.5%	+1.7%	5.3%	1.0%	−4.3%	3.7%	2.4%	−1.3%	+3.0%	−3.1%
	Medicine does not work	6.9%	17.2%	+10.3%	15.9%	15.9%	0.0%	12.6%	10.0%	−2.7%	+13.0%	+2.7%
	Taking medicine wrongly (e.g., wrong type, too much)	1.7%	6.9%	+5.2%	2.9%	1.9%	−1.0%	2.3%	2.2%	−0.1%	+5.3%	−0.9%
	Stubborn patient, medicine restrictions/dislikes	5.2%	1.7%	−3.5%	11.1%	4.8%	−6.3%	10.1%	7.5%	−2.6%	−0.9%	−3.7%
	Addicted to or strong preference for medicine	3.5%	0.0%	−3.5%	3.4%	4.3%	+1.0%	2.7%	2.7%	0.0%	−3.5%	+1.0%
	Side-effects, drug allergy, or a specific illness	13.8%	6.9%	−6.9%	7.2%	10.1%	+2.9%	8.2%	6.0%	−2.2%	−4.7%	+5.1%
	“Lueng yah”	25.9%	55.2%	+29.3%	17.3%	41.4%	+24.0%	14.7%	30.6%	+15.9%	+13.5%	+8.2%
	Other interpretation	3.5%	1.7%	−1.7%	1.9%	1.4%	−0.5%	2.1%	1.9%	−0.2%	−1.5%	−0.3%
	Don’t know / cannot or prefer not to answer	37.9%	6.9%	−31.0%	35.1%	19.2%	−15.9%	43.6%	36.8%	−6.8%	−24.2%	−9.1%
**(a2) Awareness of Drug Resistance (Lueng Yah)**	**Heard of drug resistance (“lueng yah”)**	93.1%	96.6%	+3.4%	84.6%	90.4%	+5.8%	77.5%	80.2%	+2.7%	+0.8%	+3.1%
	**Interpretations:** Reference to antibiotics / drug-resistant germs	19.0%	22.4%	+3.4%	17.8%	6.7%	−11.1%	9.6%	4.9%	−4.8%	+8.2%	−6.3%
	Medicine does not work	48.3%	44.8%	−3.5%	44.7%	53.4%	+8.7%	43.1%	41.4%	−1.6%	−1.8%	+10.3%
	Taking medicine wrongly (e.g., wrong type, too much)	3.5%	6.9%	+3.5%	2.9%	1.9%	−1.0%	2.2%	5.8%	+3.6%	−0.1%	−4.6%
	Stubborn patient, medicine restrictions/dislikes	0.0%	0.0%	0.0%	1.0%	1.0%	0.0%	1.3%	1.0%	−0.2%	+0.2%	+0.2%
	Addicted to or strong preference for medicine	20.7%	10.3%	−10.4%	21.6%	24.0%	+2.4%	20.8%	23.2%	+2.3%	−12.7%	+0.1%
	Side-effects, drug allergy, or a specific illness	1.7%	3.5%	+1.7%	0.5%	0.0%	−0.5%	1.4%	1.6%	+0.2%	+1.5%	−0.7%
	Other interpretation	3.5%	6.9%	+3.5%	2.4%	3.4%	+1.0%	4.3%	3.7%	−0.6%	+4.0%	+1.6%
	Don’t know / cannot or prefer not to answer	3.5%	5.2%	+1.7%	9.1%	9.6%	+0.5%	17.4%	18.4%	+1.0%	+0.7%	−0.6%
**(b) Attitudes and Knowledge**	**No. of desirable knowledge/attitude answers (0–4)**	1.22	1.33	+0.10	0.96	0.82	−0.14	0.90	0.79	−0.11	+0.22	−0.03
	Would buy antibiotics over the counter	48.3%	55.2%	+6.9%	32.2%	32.7%	+0.5%	28.5%	24.7%	−3.8%	+10.7%	+4.3%
	Prefers antibiotics over alternatives	32.8%	31.0%	−1.7%	29.8%	20.2%	−9.6%	28.0%	25.5%	−2.5%	+0.8%	−7.1%
	Would not keep antibiotics for future use	27.6%	32.8%	+5.2%	19.7%	28.4%	+8.7%	22.2%	26.6%	+4.4%	+0.8%	+4.3%
	Thinks that antibiotic resistance can spread	13.8%	13.8%	0.0%	14.4%	0.5%	−13.9%	11.6%	2.1%	−9.5%	+9.5%	−4.5%

Notes: Pooled data set, using matched panel data.

## Supplementary Materials

The following are available online at http://www.mdpi.com/2079-6382/7/4/95/s1, Figure S1: Changes in villagers’ recognition of the term “drug resistance” by exposure to educational activity; Figure S2: Changes in interpretations of “drug resistance” across survey rounds; Figure S3: Changes in antibiotic-related attitudes by exposure to educational activity; Table S1: Healthcare access and sources of antibiotics across survey rounds; Table S2: Sensitivity analysis: Difference in antibiotic use from formal and informal sources by respondent’s attitude towards buying antibiotics over the counter.
