# Supplementary material for: The Consequences of AMR Education and Awareness Raising: Outputs, Outcomes, and Behavioural Impacts of an Antibiotic-Related Educational Activity in Lao PDR
Source: Antibiotics (Basel). 2018 Nov 1;7(4):95. doi: 10.3390/antibiotics7040095 (PMC6316454; doi:10.3390/antibiotics7040095)
Supplement: Supplementary file 1 [file antibiotics-07-00095-s001.pdf]

Supplementary Material

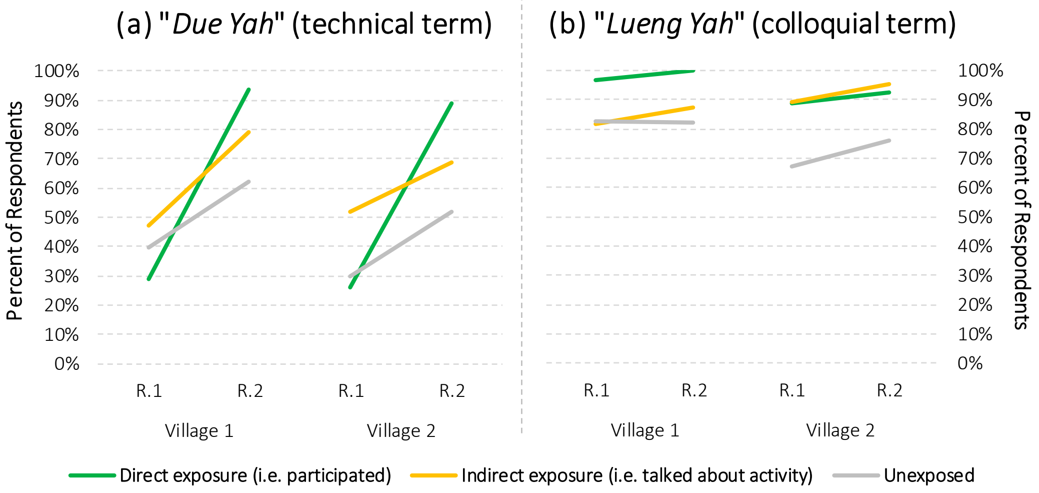

Figure S1. Changes in Villagers' Recognition of the Term "Drug Resistance" by Exposure to Educational Activity.

Notes. Pooled data set using matched panel data (n = 1,129).

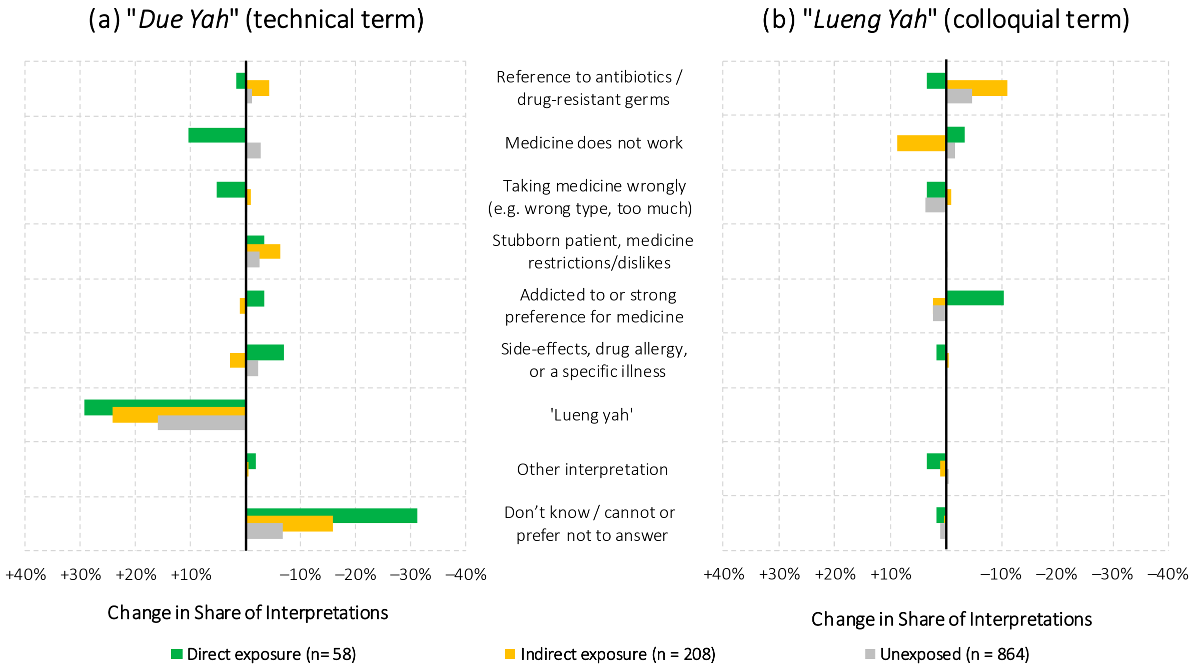

Figure S2. Changes in Interpretations of "Drug Resistance" Across Survey Rounds.

Notes. Absolute changes reported. Pooled data set using matched panel data (n = 1,129). Single response per panel.

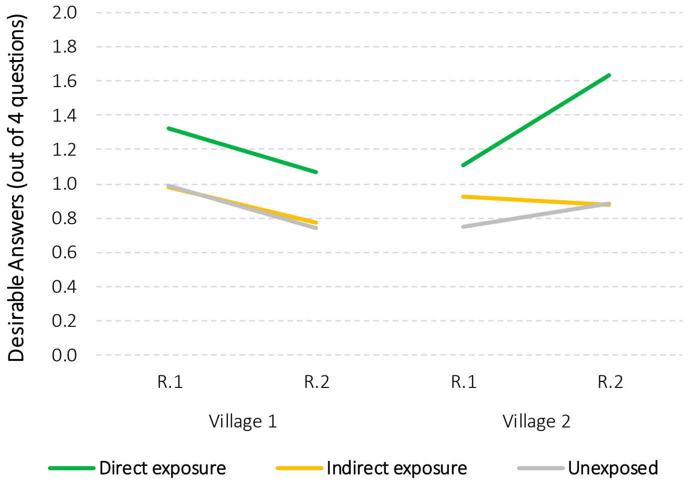

Figure S3. Changes in Antibiotic-Related Attitudes by Exposure to Educational Activity.

Notes. Pooled data set using matched panel data (n = 1,129).

Table S1. Healthcare Access and Sources of Antibiotics Across Survey Rounds.

Variable	Direct Exposure		Indirect Exposure		Unexposed	
	R.1 (n = 18)	R.2 (n = 12)	R.1 (n = 89)	R.2 (n = 58)	R.1 (n = 337)	R.2 (n = 213)
% of illness episodes involving children	39%	58%	37%	34%	43%	34%
Average self-rated severity	1.56	1.67	1.80	2.03	1.80	1.85
% public healthcare access	22.2%	50.0%	25.8%	37.9%	26.7%	22.5%
% private healthcare access	27.8%	50.0%	56.2%	53.4%	51.9%	60.6%
% informal healthcare access	0.0%	0.0%	1.1%	6.9%	2.7%	5.2%
% family and self-care	94.4%	100.0%	94.4%	100.0%	97.0%	99.5%
% other healthcare providers	22.2%	8.3%	3.4%	6.9%	6.8%	7.0%
Av. no. of non-antibiotic medicines/treatment	1.28	1.42	1.56	1.41	1.39	1.27
Av. no. of confirmed antibiotics[a]	0.72	0.75	0.55	0.57	0.49	0.36
Av. no. of confirmed & possible antibiotics (ABx)[a]	0.33	0.42	0.79	0.97	0.86	0.69
ABx from formal sources[b]	0.50	1.08	1.11	1.29	1.06	0.95
ABx from informal sources[c]	0.22	0.00	0.20	0.14	0.20	0.05

Notes. Pooled data set using complete survey data; repeated cross-sections of illness episodes (n = 727). Multiple sources of antibiotic access per illness episode possible. [a.] Based on reported medicines received during the illness and respondent's reported names and uses of antibiotics shown during the interview. [b.] Includes public and private healthcare providers. [c.] Includes traditional healers, grocery stores, retired doctors, itinerant medicine traders), and medicine stored at home and provided by family and friends.

Table S2. Sensitivity Analysis: Difference in Antibiotic Use from Formal and Informal Sources by Respondent's Attitude Towards Buying Antibiotics Over the Counter.

Variable	Undesirable Attitude	Desirable Attitude	Difference	
	Mean (Std. Dev)	Mean (Std. Dev)	Mean	p
Confirmed antibiotics from formal sources	0.36 (0.02)	0.45 (0.04)	+ 0.08	0.052
Confirmed antibiotics from informal sources	0.08 (0.01)	0.04 (0.01)	− 0.04	0.036
Confirmed & possible antibiotics from formal sources	1.02 (0.05)	1.05 (0.08)	+ 0.03	0.267
Confirmed & possible antibiotics from informal sources	0.17 (0.02)	0.07 (0.02)	− 0.10	0.003

Notes. Pooled data set using complete survey data; repeated cross-sections of illness episodes (n = 796). Hypothesis test using Wilcoxon rank-sum test.
